# Structure of catalytic domain of Matriptase in complex with Sunflower trypsin inhibitor-1

**DOI:** 10.1186/1472-6807-11-30

**Published:** 2011-06-22

**Authors:** Cai Yuan, Liqing Chen, Edward J Meehan, Norelle Daly, David J Craik, Mingdong Huang, Jacky C Ngo

**Affiliations:** 1 State Key Lab of Structural Chemistry, Fujian Institute of Research on the Structure of Matter, Chinese Academy of Sciences, Fuzhou, Fujian 350002, China; 2 Laboratory for Structural Biology, Department of Chemistry, University of Alabama in Huntsville, Huntsville, AL 35899, USA; 3 University of Queensland, Institute for Molecular Bioscience, Division of Chemistry and Structural Biology, Brisbane, Queensland 4072, Australia; 4 The Chinese University of Hong Kong, Shatin, Hong Kong SAR, China

## Abstract

**Background:**

Matriptase is a type II transmembrane serine protease that is found on the surfaces of epithelial cells and certain cancer cells. Matriptase has been implicated in the degradation of certain extracellular matrix components as well as the activation of various cellular proteins and proteases, including hepatocyte growth factor and urokinase. Sunflower trypsin inhibitor-1 (SFTI-1), a cyclic peptide inhibitor originally isolated from sunflower seeds, exhibits potent inhibitory activity toward matriptase.

**Results:**

We have engineered and produced recombinant proteins of the matriptase protease domain, and have determined the crystal structures of the protease:SFTI-1 complex at 2.0 Å as well as the protease:benzamidine complex at 1.2 Å. These structures elaborate the structural basis of substrate selectivity of matriptase, and show that the matriptase S1 substrate specificity pocket is larger enough to allow movement of benzamidine inside the S1 pocket. Our study also reveals that SFTI-1 binds to matriptase in a way similar to its binding to trypsin despite the significantly different isoelectric points of the two proteins (5.6 vs. 8.2).

**Conclusions:**

This work helps to define the structural basis of substrate specificity of matriptase and the interactions between the inhibitor and protease. The complex structure also provides a structural template for designing new SFTI-1 derivatives with better potency and selectivity against matriptase and other proteases.

## Background

Matriptase is a type II transmembrane serine protease of the S1 trypsin-like family. Matriptase activity is down-regulated by its physiological inhibitor, hepatocyte growth factor activator inhibitor-1 (HAI-1) [1-3]. Matriptase is expressed in most epithelial cells and plays essential roles in the establishment and maintenance of epithelial integrity. New evidence suggests that matriptase is also expressed on mast cells, peripheral blood monocytes and B cells, implicating matriptase in the physiological and pathologic functions of these cells [4-6]. Knock down studies in mice have shown that the protease is important in postnatal survival, epidermal barrier formation, hair follicle growth and thymichomeostasis [7]. At the same time, genetic studies using zebra fish and mice have indicated that the activity of matriptase is critical for tissue-integrity and function, and must be strictly controlled by HAI-1 [8-11].

The catalytic domain of matriptase is tethered to the cell surface via its N-terminal signal anchor, linked by a sea urchin sperm protein/enterokinase/agrin (SEA) domain, two tandem complement/urchin embryonic growth factor/bone morphogenetic protein (CUB) domains, and four tandem low-density lipoprotein receptor class A (LDLRA) domains. Interestingly, matriptase activation does not depend on other active proteases. Instead, several lines of evidence have indicated that matriptase undergoes autoactivation through a mechanism relying on its own catalytic triad and requires its non-catalytic domains as well as the presence of its cognate inhibitor HAI-1 [12,13]. Although the autoactivation mechanism is not fully understood, one study has showed that matriptase could be activated by acidification, and suggested that matriptase might act as an early response to cellular acidosis [14]. Once activated, matriptase has only short time to cleave and activate its substrates since the protease will be quickly inhibited by HAI-1.

Matriptase activates a number of substrates, including G-protein-coupled protease-activated receptor 2, urokinase plasminogen activator and pro-hepatocyte growth factor [15,16]. Recently, it has been demonstrated that matriptase could also activate prekallikren either *in vitro *or *in vivo *[17]. Matriptase is recognized as a cancer-associated protease since the activation of urokinase plasminogen activator and/or pro-hepatocyte growth factor has been implicated in cancer invasion and metastasis (reviewed in [18]). In addition, matriptase has been found to be upregulated in various forms of cancers including breast, cervical, ovarian, liver, and prostate cancers. It has been demonstrated that the level of expression of matriptase correlates with the tumor stage and malignancy of breast, cervical, ovarian and prostate cancers [19-21]. In some of these cancers, the ratios of the protease relative to its inhibitor HAI-1 are unbalanced; suggesting that strict regulation of matriptase by HAI-1 is required to prevent carcinogenesis. A recent study showed that matriptase orthotopically overexpressed at modest levels in the skin of transgenic mice caused spontaneous squamous cell carcinoma, potentiated chemical carcinogenesis, and supported both ras-dependent and -independent carcinogenesis, whereas the overexpression of HAI-1 could nullify these oncogenic effects [22]. In addition to its role in cancers, recent studies have suggested that matriptase also has potential implications in a variety of diseases including osteroarthritis, atherosclerosis, and skin disorders like autosomal recessive ichthyosis and hypotrichosis (ARIH) [4,23-26]. Taken together, matriptase has emerged as an attractive target for the development of anti-metastasis therapy as well as treatment for many other diseases.

Sunflower trypsin inhibitor-1 (SFTI-1), a 14-amino acid cyclic peptide, is originally isolated from sunflower seeds and characterized as the most potent peptidic inhibitor of trypsin (Ki = 0.1 nM and 1 nM from two independent studies) [27,28]. A later study finds that synthetic SFTI-1 also exhibits very potent matriptase inhibitory activity (Ki = 0.92 nM) [27]. To evaluate the structural basis of the high inhibitory effect of SFTI-1 to matriptase, we have determined the X-ray structure of matriptase in complex with SFTI-1. We have also determined the high-resolution structure of matriptase:benzamidine complex for structural comparison. The crystal structures provide new insights into the molecular basis of matriptase inhibition and this information might facilitate future design of more potent and selective peptide inhibitors using SFTI-1 as template.

## Results and Discussion

### Engineering of recombinant matriptase catalytic domain in P. pastoris for structural study

For our structural studies, we constructed a recombinant protease domain of matriptase (residue 615 to 854 of the EXPASY entry Q9Y5Y6) with a point mutation N164Q (chymotrypsin numbering will be used throughout the paper starting from here), which is referred as β-matriptase-N164Q. The point mutation removes a glycosylation site (N164) and allows the protein to be purified to homogeneity. Another unique feature of the current design of the expression scheme is that the secreted recombinant matriptase protease domain is an active serine protease without the need of being activated. This is due to the processing of the secreted protein by an endogenous kex2 enzyme of *P. pastoris *that generates the genuine N-terminus of matriptase protease domain and allows the correct folding of the protease into its active form. We have used such approach to generate a number of active proteases including urokinase-type plasminogen activator [29], tissue-type plasminogen activator and coagulation factor XIa (to be published), suggesting that our method can be widely adapted for the expression of different active proteases.

### Structure of β-matriptase-N164Q:benzamidine shows benzamidine mobility

**Figure 1:**
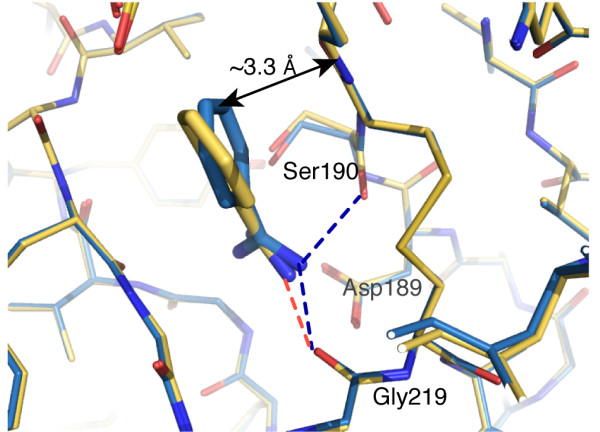
**Comparison of benzamidine orientations in two matriptase:benzamidine complexes**. Benzamidine mediates extra hydrogen bonding and van der Waals interactions to the peptide segment of Ser190-Gln192 in our structure (blue) when compared to 1EAX (yellow).

In the structure of β-matriptase-N164Q complexed with benzamidine, the benzamidine binds to the specificity pocket S1 right next to the active site and is sandwiched by the segments of Ser190-Gln192 and Trp215-Gly216 as expected. This structure is similar to the previously reported structure of the matriptase protease domain in complex with benzamidine (PDB ID = 1EAX, RMSD = 0.1 Å for the Cα atoms of 227 residues) [30]. However, there are interesting differences between these two structures. The phenyl group of the benzamidine in our structure shows a 35° rotation when compared to the 1EAX structure. While the amidino group of the inhibitor juxtaposes the side chain of Asp189 and forms favorable ionic interaction with its carboxylate group as in the 1EAX structure, the bis-amine in our structure is rotated for ~20° and makes an extra hydrogen bond with the backbone carbonyl of Ser190, in addition to those formed with the Gly219 carboxylate and a conserved solvent molecule in 1EAX (Figure 1). Considering the high resolutions of both matriptase-benzamidine structures (1.2 Å in our structure) and the relatively small errors of structures (0.4 Å in our structure), such differences in benzamidines are significant, and indicates that S1 pocket is larger than the molecular size of benzamidine and allows movement of benzamidine inside the S1 specificity pocket. This is consistent with the observed higher temperature factor of benzamidine (40 Å^2^) comparing to the average temperature factor of protein (15 Å^2^).

### Interactions between matriptase and SFTI-1 at the active site

**Figure 2:**
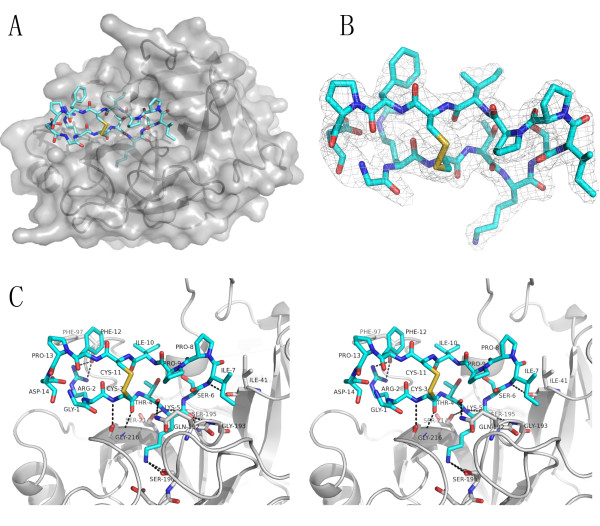
**Crystal structure of matriptase:SFTI-1 complex**. A) Overall structure of SFTI-1-bound matriptase in surface rendition (grey) with SFTI-1 in cyan. B) SFTI forms a bicyclic structure. 2Fo-Fc electron density map of the peptide inhibitor contoured at 1σ is shown. C) Interactions of matriptase (grey) and SFTI-1 in stereo view. Intermolecular hydrogen bonds formed between the protease and inhibitor are denoted by dashed lines.

Since SFTI-1 is a highly potent inhibitor to either matriptase or trypsin with comparable potencies, we want to investigate if the inhibitory mechanisms are the same for the two proteases. The crystal structure of β-matriptase-N164Q in complex with SFTI-1 was solved at 2.0 Å (Figure 2A). In this structure, SFTI-1 forms a bicyclic structure where two antiparallel β-strands are constrained by a disulfide bond between Cys3 and Cys11 (Figure 2B). The cyclic peptidic inhibitor docks onto matriptase's P4-P2' specificity sites with good shape and charge complementarity. This resembles the SFTI-1 binding to trypsin and other BBI to serine proteases, as well as the reactive site loop of the bovine pancreatic trypsin inhibitor (BPTI) to matriptase [28,30-33]. Like the equivalent side chains in the other complexes, Lys5 from the "reactive loop" of SFTI-1 extends into the S1 pocket of matriptase to form a hydrogen bond directly with Ser190 and may also interact with Asp189 at the bottom of the pocket (Figure 2C and Table 1; Table 1 summarizes hydrogen bonds formed between matriptase and SFTI-1). The SFTI-1 scissile bond (Lys5-Ser6) is in close proximity to the catalytic residue Ser195 where the Ser195 hydroxyl group hydrogen bonds with the main-chain amide of Ser6 and makes close contacts with the backbone carbonyl and amide of Lys5 (2.9Å and 3.0 Å respectively). The side chain oxygen atom of Ser195 is also in close contact with the carbonyl carbon of P1 residue (Lys5) (2.6 Å) at an angle of 88.6° (Lys5O-C-Ser195OG), suggesting the protease is in an active conformation capable of initiating bond scission. Furthermore, His57 is suitably oriented to interact with Ser195 to support catalysis (at 2.6 Å and with Ser195CB-OG-His57NE2 angle of 101.4°). These observations support that SFTI-1 functions as a substrate variant of matriptase in a similar manner to other BBIs. Thus, the cyclic peptidic inhibitor is likely to inhibit the enzyme through high affinity binding to the active site and through excluding water molecules from the active site to destabilize the reaction-path transition state.

**Table 1:** Hydrogen bonds between matriptase (chymotrypsin numbering) and SFTI-1

SFTI-1 residue/atoms	Matriptase residue/atoms	Distance (Å)
Arg2 NH1	Phe97 O	2.8
Arg2 NH2	Phe97 O	2.9
Cys3 N	Gly216 O	3.0
Cys3 O	Gly216 N	3.0
Thr4 O	Gln192 NE2	3.0
Lys5 N	Ser214 O	3.2
Lys5 NZ	Ser190 OG	2.9
Lys5 O	Ser195 N	2.9
Lys5 O	Gly193 N	2.8
Ser6 N	Ser195 OG	3.0
Ile7 N	Ile41 O	3.3

Comparison of the conformations of matriptase in the benzamidine- and SFTI-1-bound forms reveals that they are nearly identical except the side chain of Phe99 at the S2 subsite, which will be discussed later, and Gln192 at the S3 subsite. Gln192 side chain is solvent-exposed in the benzamidine-bound structure and forms the lining of the extended active site groove. However, it undergoes a major conformational change to "bend inward" to accommodate SFTI-1 and hydrogen bonds with the backbone carbonyl of Thr4 of the cyclic inhibitor in a manner similar to that in the BPTI-bound matriptase structure.

### Substrate selectivity of matriptase

**Figure 3:**
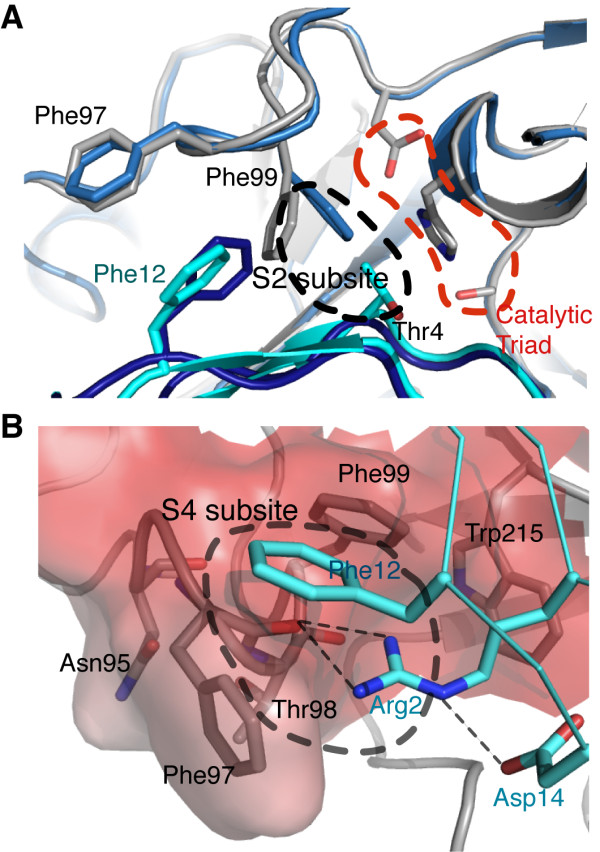
**Comparison of matriptase:SFTI-1 and trypisn:SFTI-1 complexes**. A) The matriptase Phe99 side chains (grey) and SFTI Phe12 -1 (cyan) undergo large rotation to widen the S2 subsite when compared to the structures of matriptase in benzamidine-bound form (light blue) and trypsin: SFTI-1 complex (deep blue). B) SFTI-1 Arg2 is accommodated at the S4 subsite (in surface representation and colored by electrostatic potential, ±7 kT/e).

Previous studies using positional scanning-synthetic combinatorial library and substrate phage display have revealed that matriptase also strongly prefers substrates with small residues including glycine and serine, or phenylalanine at the P2 position, in addition to the protease specificity for basic residue at the P1 pocket [16]. Moreover, the most effective substrate has been found to contain basic residues at the P3 or P4 sites, but not both simultaneously [16]. Indeed, the sequence of SFTI-1 matches these findings except the presence of a threonine at the P2 position. Our structure explains how SFTI-1 achieves such substrate selectivity. In the structure, the S2 subsite of matriptase is occupied by SFTI-1 Thr4 with Thr4 side chain flanked by His57 and Phe99. The methyl group of Thr4 makes CH-π interaction with both flanking side chains and docks snugly in the pocket. Comparing to the benzamidine-bound structure, we observed that the benzyl ring of Phe99 undergoes a rotation to widen the pocket to better accommodate the P2 residue (Figure 3A). Similar observation has also been made in the BPTI:matriptase complex [30]. This structural explanation for the suitability of threonine at P2 position suggests that the S2 pocket is not rigid and its size is controlled by the conformation of Phe99. A large residue at substrate P2 position will inevitably cause steric hindrance with His57 and alter its position, and will disrupt the hydrogen bond between Asp102 and His57 and hinder catalysis. Yet, despite its large size, a phenylalanine at P2 would make favorable π-π interactions with Phe99 and His57, and/or cation-π interaction when the histidine is protonated during catalysis, explaining why phenylalanine is also preferred in addition to small residues at this site [16].

Comparison with the trypsin:SFTI-1 complex (PDB ID = 1SFI) reveals that the β-turn consisted of Phe12, Pro13, Asp14 and Gly1 of SFTI-1 adopts a slightly different conformation upon binding to matriptase [27,28]. This is to avoid steric hindrance with the matriptase 99 loop. Particularly, both side chains of SFTI-1 Phe12 and matriptase Phe99 (Leu99 in trypsin) undergo large rotational changes to avoid clashing with each other (Figure 3A). In its new conformation, Phe12 is now stabilized by van der Waals interaction with the benzyl rings of Phe97 and Phe99. Gly1 consequently shifts slightly closer to the active site groove by ~0.6 Å, allowing its main-chain carbonyl to make an extra contact with the amide of Gly219. Interaction between Asp14 and Gln175 of trypsin is replaced with intramolecular interaction between Asp14 and Arg2 of SFTI-1, which stabilizes the conformation of Arg2 side chain to interact with the S4 subsite (Figure 3B). The backbone carbonyls of Asp95, Phe97 and Thr98 form the S4 subsite, which is confined and protected from the solvent by the side chains of Phe97 and Phe99 [30]. Previous positional scanning and substrate phage display studies performed by Takeuchi *et al *[16] had revealed that the S4 subsite of matriptase strongly prefers basic residues. Our structure provides a clear explanation on this selectivity. Firstly, unlike in the trypsin-SFTI-1 complex, the guanidinium group of Arg2 is anchored by Asp14 to form two hydrogen bonds with the carbonyl of Phe97. Secondly, the rearrangement of the benzyl groups of Phe97 and Phe99 results in a solvent-shielding environment that lowers the dielectric constant and thus stabilizes the hydrogen bonding interactions mediated by Arg2 (Figure 3B). More importantly, the guanidinium group of Arg2 forms cation-π interactions with both phenylalanines as well as Trp215 in T-shaped geometries and provides further stabilization [34,35]. To confirm these interactions, we used the program CaPTURE to estimate the stabilizing energies from the cation-π interactions (Table 2) [34]. The interaction between Arg2 and the S4 subsite alone is estimated to contribute ~12 kcal/mol, suggesting that the S4 subsite may play a key role in matriptase specificity. Indeed, inhibitory assays with different SFTI-1 derivatives have shown that the S4 subsite strongly prefers arginine [36]. Minor modifications of the Arg2 guanidinium in SFTI-1 result in significant loss of matriptase inhibition, and replacement of Arg2 with a Phe(Glu) decreases the inhibitory activity by 900-fold [36].

**Table 2:** Calculated binding energies of cation-π interactions between matriptase and SFTI-1 [34]

SFTI-1 side chain	Matriptase aromatic side chain	E(electrostatic) (kcal/mol)	E(van der Waals) (kcal/mol)
Arg2	Phe97	-2.86	-1.17
Arg2	Phe99	-2.01	-1.19
Arg2	Trp215	-3.76	-1.01

### Comparison of SFTI-1 binding to matriptase and trypsin

**Figure 4:**
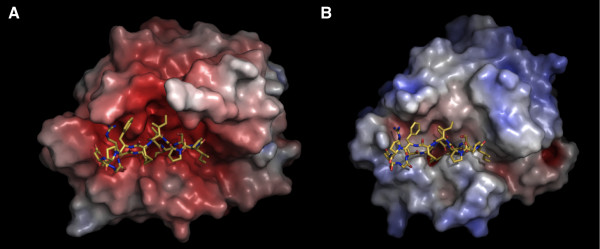
**Solvent-accessible surfaces of (A) matriptase and (B) trypsin are colored by electrostatic potential (-7 to +7 kT/e in blue to red) computed by APBS **[48]. The matriptase active site surface is more acidic than that of trypsin.

Matriptase protease domain contains many acidic residues, leading to a lower theoretical pI in comparison with bovine trypsin (5.6 vs. 8.2). In addition, the electrostatic surface potential of the active site groove of the matriptase protease domain is much more negatively charged than that of trypsin (Figure 4A and 4B). Thus, the basic SFTI-1 (pI = 8.1) would presumably favor matriptase binding than trypsin binding. However, SFTI-1 has been shown to inhibit trypsin and matriptase with comparable affinities [27], and we observed here that the binding interface of SFTI-1 to matriptase is structurally very similar to trypsin. Such apparent discrepancy may be due to the lower mobility of SFTI-1, and thus stronger binding, in the current matriptase:SFTI-1 crystal structure when compared to the trypsin:SFTI-1 structure (relative mobility of 0.7 vs. 1.5). Here the relative mobility is defined by the average temperature factor of SFTI-1 in the structure of the complexes divided, by the average temperature factor of the rest of protein in order to cancel out the effect of resolution on the temperature factor. Another possible explanation is to take into account the penalty of the loss of configurational entropy of both protein and ligand upon SFTI-1 binding [37]. For instance, Phe12 of SFTI-1 is more constrained in the matriptase complex than in the trypsin complex due to the presence of Phe97 and Phe99 that surround it, which might result in a lower torsional degree of freedom and yield an entropy penalty, compensating the gain of enthalpy from the charge complementarity.

### Implication for future inhibitor design

The current structure provides a template for further improvement of SFTI-1. For instance, non-polar residues like Ile7 and Ile10 are good candidates for modifications of this bicyclic peptide to improve its binding enthalpy. Ile10 is located in a cavity formed by the surface exposed insertion loops (loops 66 and 99) of matriptase and is proximal to the active site. However, it does not make any direct contacts with residues from the protease. Previous work by Li *et al. *shows that Ile10 plays an important role in the selectivity of the inhibitor as its replacement by a more polar and bulky glutamine improves the compound selectivity for matriptase versus thrombin by >1073 fold [36]. Based on the electrostatic surface potential of matriptase calculated from our structure, the modification of SFTI-1 Ile10 to a positively charged amino acid might fit the cavity more tightly and provide a favorable enthalpy. However, increasing flexibility of the inhibitor might result in loss of configurational entropy upon binding, as illustrated by the higher K_i _of the Gln10 derivative of SFTI-1 [36]. Therefore, flexible amino acids like lysine and arginine should be avoided for the substitution and small positively charged unnatural amino acids such as diaminopropionic acid or diaminobutyric acid may be used instead. These small side chains will likely favor the interaction with Asp96 without disrupting the conformation of the catalytic triad and improves the enthalpy of binding while minimizing the entropy penalty [38]. Similarly, Ile7 lies on top of a groove adjacent to the catalytic cleft of matriptase and its sole direct interaction with matriptase is through weak van der Waals interaction with Ile41. We believe our suggested strategy for the modification of Ile10 can also be applied to Ile7. Together, the combination of modifications at Ile7 and Ile10 should improve the inhibitory activity of SFTI-1 towards matriptase.

## Conclusions

SFTI-1 is originally isolated and characterized as a potent trypsin inhibitor. It has also been synthesized by Roller's group and shown to exhibit potent inhibitory effect against matriptase. The same group also investigated the structural basis of the high inhibitory activity of SFTI-1 using molecular modeling study and obtained information that aids the design and synthesis of new SFTI-1 analogs. While modification to stabilize the disulfide bond within the cyclic peptide maintains the compound's inhibitory potency and selectivity of matriptase versus thrombin, replacement of Ile10 with the more polar glutamine improves selectivity towards matriptase at the expense of weakening its inhibitory activity. Nevertheless, none of the modified inhibitors show improvement in binding affinity to matriptase. This major drawback can now be overcome by better aid from the structural information of an experimentally obtained structure of matriptase:SFTI-1 complex. This work helps to define the structural basis of substrate specificity of matriptase and provides more details in the interactions between the inhibitor and protease. Our structure also reveals the structural difference between the SFTI-1 bound matriptase and trypsin complexes to allow development of more potent and selective inhibitors for matriptase.

## Methods

### Recombinant protein expression and Purification

The matriptase N164Q (chymotrypsin numbering) catalytic domain mutant was first generated by site-directed mutagenesis using cDNA encoding the entire protease domain of human matriptase as template. The cDNA was then amplified by PCR using primers containing XhoI and SalI restriction sites. The purified PCR products were digested with XhoI and SalI and subcloned into the XhoI-SalI sites of the *Pichia pastoris *(*P. pastoris*) expression vector pPICZαA (Invitrogen). Plasmid DNAs were linearized with the restriction enzyme SacI prior to transformation into *P. pastoris *strain X-33. Recombinant matriptases were expressed in *P. pastoris *according to the manufacturer's recommendations. *P. pastoris *expression medium was concentrated 10-20 fold using a Millipore concentrator (8000 Da MWCO membrane) and pH was adjusted to 7.4. The concentrated medium was applied onto a benzamidine column (GE Healthcare) equilibrated with 50 mM Tris, 0.5 M NaCl, pH7.4, and eluted with 100 mM glycine, pH 3.0. The elution fractions were neutralized with 1 M Tris pH 9 immediately. Fractions containing matriptase activity were pooled and concentrated, and passed through MonoQ column (Amersham Biosciences, Inc.) pre-equilibrated with 40 mM Tris. The protein was eluted in a buffer containing 40 mM Tris, pH7.4 with a 0-0.4 M NaCl gradient. Fractions containing protein were pooled and concentrated to 5 mg/ml. Aliquots of the purified protein were frozen at 193 K for crystallization experiments.

### Crystallization of β-matriptase-N164Q complex with its inhibitors

For protein crystallization, *β-matriptase-N164Q:benzamidine *complexes were mixed at 1:10 ratio, and crystallized by the hanging-drop vapor diffusion method with a precipitant solution of 0.1 M Tris, pH 8.0, 1.5 M ammonium sulfate, 3% ethanol. SFTI-1 was synthesized by solid state synthesis as previously reported [39]. The complex of β-matriptase-N164Q with SFTI-1 was crystallized with a precipitant condition of 22% polyethylene glycol 8000, 0.1 M Tris pH 8 and 20 mM CaCl_2_.

### Data collection, structure solution and refinement

X-ray diffraction data were collected at APS to 1.2 Å and 2.0 Å for β-matriptase-N164Q:benzamidine and β-matriptase-N164Q:SFTI-1 complexes, respectively (Table 3). The crystal structures of the complexes were solved using the program MOLREP [40] from the CCP4 program suite [41] and the published β-matriptase structure (Protein Data Bank (PDB) ID: 1EAX) [30] as the search model. After refinement using the REFMAC program [42], the Fo-Fc difference electron density showed extra electron density attaching to Cys122. Based on the contour of this extra electron density, a molecule of reduced glutathione (GSH) was modeled covalently linking to Cys122 (Additional file 1). In the structure of β-matriptase-SFTI-1 complex, SFTI-1 was modeled into the extra electron density around matriptase active pocket. The resulting models were refined using Phenix [43], and manual model fitting was carried out using the program COOT [44]. In the final cycles of refinement, TLS & restrained refinement with twenty TLS groups which generated by the TLS motion determination (TLSMD) server [45] was applied. Detailed refinement and structure statistics are listed in Table 3. The final structures were analyzed by PROCHECK [46] and PYMOL [47].

**Table 3:** Statistics of X-ray diffraction data collection and structure refinement

	β-matriptase-N164Q:benzamidine	β-matriptase-N164Q:SFTI-1
Diffraction data		
Space group	C222	P41212
Cell parameters (Å)	66.9, 141.7, 52.0	75.9, 75.9, 94.1
R_merge _(%)^a^	0.1 (0.5)^a^	0.1 (0.5)^a^
Completeness (%)	91.6 (94.6)^a^	98.9 (100.0)^a^
Average I/σ	18.3 (1.5)^a^	38.5 (3.0)^a^
Data redundancy	5.7 (2.9)^a^	12.1 (11.1)^a^
Refinement		
Resolution (Å)	70.9-1.2	25.8-2.0
R_work_/R_free _(%)	17.8/19.9	19.4/24.5
RMSD		
Bond length (Å)	0.006	0.003
Bond angles (°)	1.20	0.70
Mean B factors (Å^2^)	12.5	50.7
Ramachandran plot, % residues in regions:		
Most favored	97.5	96.4
Additionally allowed	2.5	3.6
Generously allowed	0	0
Disallowed	0	0
PDB ID Code	3P8G	3P8F

^a^Numbers in the parentheses are for the highest resolution shell.

## Abbreviations

BBI: Bowman-Birk inhibitor; BPTI: bovine pancreatic trypsin inhibitor; HAI-1: hepatocyte growth factor activator inhibitor-1; PDB: Protein Data Bank; RMSD: root mean square deviation; SFTI-1: Sunflower trypsin inhibitor-1.

## Authors' contributions

MH designed the study, MH and JCN wrote and revised the manuscript, CY participated in the design of the study, carried out all experiments in molecular biology, protein chemistry, structure refinement and drafted the manuscript, ND and DJ synthesized SFTI-1 peptide, LC and EJM collected the X-ray data, and all authors read and approved the final manuscript.

## Supplementary Material

Additional file 1 **Fig. S1**. The extra electron density (in stereo representation) around the Cys122 of the β-matriptase-N164Q:benzamidine structure suggests the Cys122 is conjugated by a disulfide bond (yellow) to a glutathione (GSH, in green sticks). GSH might have attached to the protein during protein synthesis because GSH is one of the abundant intracellular sulfhydryl antioxidants in yeast as a similar observation was made in other structures including the rhFXI-benzamidine complex 49 where the protein was also expressed in *P. pastoris *. 2Fo-Fc electron density map is contoured at 1s at 1.2 Å. Click here for file
