# Supplementary figures and images for: Structure of catalytic domain of Matriptase in complex with Sunflower trypsin inhibitor-1
Source: BMC Struct Biol. 2011 Jun 22;11:30. doi: 10.1186/1472-6807-11-30 (PMC3141381; doi:10.1186/1472-6807-11-30)

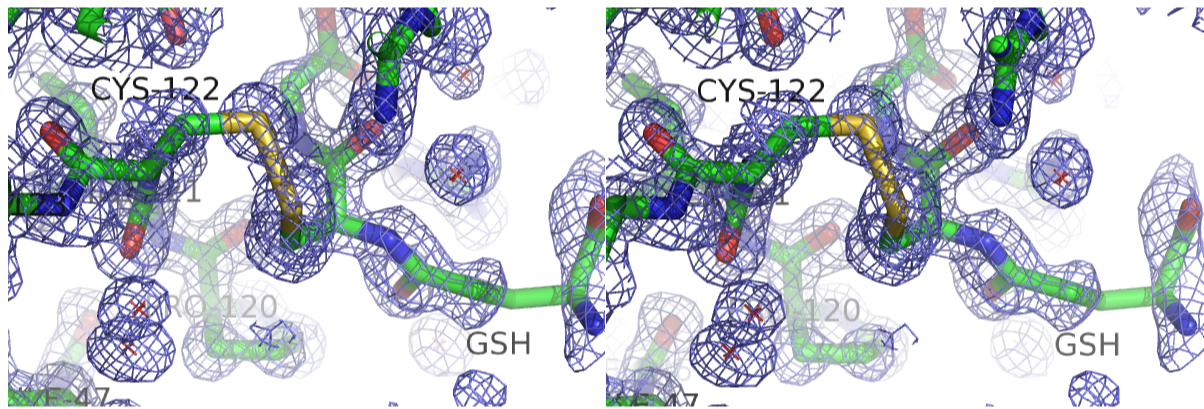

Supplement: Additional file 1 — Fig. S1. The extra electron density (in stereo representation) around the Cys122 of the β-matriptase-N164Q:benzamidine structure suggests the Cys122 is conjugated by a disulfide bond (yellow) to a glutathione (GSH, in green sticks). GSH might have attached to the protein during protein synthesis because GSH is one of the abundant intracellular sulfhydryl antioxidants in yeast as a similar observation was made in other structures including the rhFXI-benzamidine complex 49 where the protein was also expressed in P. pastoris . 2Fo-Fc electron density map is contoured at 1s at 1.2 Å. [file 1472-6807-11-30-S1.TIFF]
